# STIP1/HOP Regulates the Actin Cytoskeleton through Interactions with Actin and Changes in Actin-Binding Proteins Cofilin and Profilin

**DOI:** 10.3390/ijms21093152

**Published:** 2020-04-30

**Authors:** Samantha Joy Beckley, Morgan Campbell Hunter, Sarah Naulikha Kituyi, Ianthe Wingate, Abantika Chakraborty, Kelly Schwarz, Matodzi Portia Makhubu, Robert Pierre Rousseau, Duncan Kyle Ruck, Jo-Anne de la Mare, Gregory Lloyd Blatch, Adrienne Lesley Edkins

**Affiliations:** 1 Biomedical Biotechnology Research Unit, Department of Biochemistry and Microbiology, Rhodes University, Grahamstown 6140, South Africa; sambeckley@gmail.com (S.J.B.); morgan.hunter@pharma.ethz.ch (M.C.H.); skituyi@gmail.com (S.N.K.); ianthe.wingate@gmail.com (I.W.); abanbubli90@gmail.com (A.C.); kellyschwarz0@gmail.com (K.S.); pmaumela@gmail.com (M.P.M.); robrss.9641@gmail.com (R.P.R.); duncan.ruck@gmail.com (D.K.R.); j.delamare@ru.ac.za (J.-A.d.l.M.); g.blatch@ru.ac.za (G.L.B.); 2 Centre for Chemico- and Biomedicinal Research, Rhodes University, Grahamstown 6140, South Africa; 3 The Vice Chancellery, The University of Notre Dame Australia, Fremantle, WA 6160, Australia

**Keywords:** *STIP1*, *STI1*, HOP, actin, ATPase activity, profilin, cofilin

## Abstract

Cell migration plays a vital role in both health and disease. It is driven by reorganization of the actin cytoskeleton, which is regulated by actin-binding proteins cofilin and profilin. Stress-inducible phosphoprotein 1 (STIP1) is a well-described co-chaperone of the Hsp90 chaperone system, and our findings identify a potential regulatory role of STIP1 in actin dynamics. We show that STIP1 can be isolated in complex with actin and Hsp90 from HEK293T cells and directly interacts with actin in vitro via the C-terminal TPR2AB-DP2 domain of STIP1, potentially due to a region spanning two putative actin-binding motifs. We found that STIP1 could stimulate the in vitro ATPase activity of actin, suggesting a potential role in the modulation of F-actin formation. Interestingly, while STIP1 depletion in HEK293T cells had no major effect on total actin levels, it led to increased nuclear accumulation of actin, disorganization of F-actin structures, and an increase and decrease in cofilin and profilin levels, respectively. This study suggests that STIP1 regulates the cytoskeleton by interacting with actin, or via regulating the ratio of proteins known to affect actin dynamics.

## 1. Introduction

Cell migration is vital for embryonic morphogenesis, tissue repair and regeneration, the immune response and in progression of diseases such as cancer [1,2]. Migration of cells is driven by the reorganization of the cytoskeleton comprised of tubulin and actin filaments [3,4,5]. Actin is largely responsible for driving cell motility by pushing the cell membrane in the direction of migration, forming protrusions at the leading edge of the cell [1,6]. There are two forms of actin present in the cell—the monomeric globular form otherwise known as G-actin, which has a molecular mass of 42 kDa and is soluble, and the polymerised, filamentous form, F-actin, which makes up the helical microfilaments of the cytoskeleton [6,7]. One end of the formed filament is known as the minus end or the pointed end, whereas the other end is known as the plus end, fast growing end or the barbed end [6,8]. Addition of actin monomers can occur at both ends but is favoured at the plus end. Disassembly of actin filaments is triggered by hydrolysis of ATP and a relatively slow dissociation of the phosphate [9,10]. ATP-actin associates at the barbed end and ADP-actin disassociates from the pointed end in an essential process for migration known as actin treadmilling [9]. In addition to the ATPase activity of actin, F-actin formation, and hence cell migration, is regulated by protein–protein interactions [9,11], involving a core group of actin-binding proteins that include cofilin and profilin. Cofilin interacts with F-actin to promote depolymerisation, while profilin functions to promote filament formation [12,13,14]. Profilin and cofilin regulate actin polymerisation [15,16,17] and localization [18]. Profilin promotes the polymerisation of G-actin into F-actin filaments, whilst cofilin is responsible for disassembly of actin fibres [19,20]. Cofilin can modulate the rate of actin polymerisation/depolymerisation by its actin-severing capability [21,22,23] and has been shown to play a major role in metastasis and invasiveness in breast cancer [24]. Profilin expression was suppressed in cancer cells [25,26] and silencing of profilin in human umbilical vein endothelial cells (HUVEC) resulted in decreased cell migration in wound healing assays [27,28]. In the nucleus, actin functions in chromatin remodelling and organization, as well as regulating transcription complexes and ribonucleoproteins [29,30,31,32,33,34,35]. Many of the actin regulatory proteins including cofilin and profilin are also found in the nucleus, where they regulate polymerisation and localization of actin. Cofilin has a nuclear localization signal (NLS) and shuttles into the nucleus under stress, promoting rod-like structures of actin [36,37,38]. Cofilin is required for nuclear import of actin and for maintaining the monomeric nuclear actin pool [18]. Profilin is predominantly nuclear and acts as a cofactor with exportin-6 in the export of actin out of the nucleus [39,40].

Molecular chaperones are responsible for the maintenance of protein stability and protein translocation in the cell [41,42]. Certain members of the heat shock protein (Hsp) family function as molecular chaperones for proteins including those of the cytoskeleton. Hsp90 binds to actin in vitro [43] and regulates bundling of actin filaments via N-WASP and Arp2/3 complexes [44]. Transient colocalization of Hsp70 and actin has been reported after heat shock treatment in *Dictyostelium* [45]. A direct interaction between small Hsps (sHsps) and actin occurs, which inhibits actin polymerisation and confers protection to the microfilaments by binding of phosphorylated sHsp oligomers to F-actin [46]. The Bcl-2-associated athanogene 3 (BAG3), along with a small Hsp (HSPB8), performs a role in proteostasis in mechanical stress, and knockdown of BAG3 and HSPB8 led to defects in the actin-based remodelling system and spindle orientation in mitotic cells [47]. BAG3 is part of the family of co-chaperones, which act as non-client chaperone-binding proteins and assist in the functioning of the chaperones [48]. Hsp70 and Hsp90 share a co-chaperone termed the Hsp70/Hsp90-organizing protein (HOP), also known as stress-inducible phosphoprotein 1 (STI1/STIP1) [49], the knockout of which is embryonic lethal in the mouse [50]. STIP1 is overexpressed in several cancer types, where high expression served as a biomarker and correlated with reduced survival and increased metastasis [51,52,53,54,55,56]. Recombinant STIP1, as well as microglial-secreted STIP1, has been demonstrated to promote cell migration of glioblastoma cells [57]. It was speculated that this increase in cell migration occurs via the modulation of matrix metallopeptidase 9 (MMP-9), which is able to degrade the extracellular matrix [57,58]. This was confirmed by a decrease in MMP-9 activity in the presence of an anti-STIP1 antibody [57]. In addition to these extracellular functions, our laboratory showed colocalization between intracellular STIP1 and the actin pathway signalling protein, RhoC. In addition, STIP1 and actin colocalized at the leading edges of pseudopodia in breast cancer cells and knockdown of STIP1 resulted in decreased levels of RhoC and a reduction in pseudopodia formation. [59]. Using an actin co-sedimentation assay, we further demonstrated that STIP1 and actin interacted in vitro. These data suggested that STIP1 may play a role in migration by interacting with actin during the reorganization of the actin cytoskeleton [59]. Here, we extend the analysis of the STIP1–actin interaction and describe how the interaction of STIP1 with actin has implications for cell migration.

## 2. Results and Discussion

### 2.1. mSTI1 Interacts with F-actin via the TPR2AB Domain

**Figure 1:**
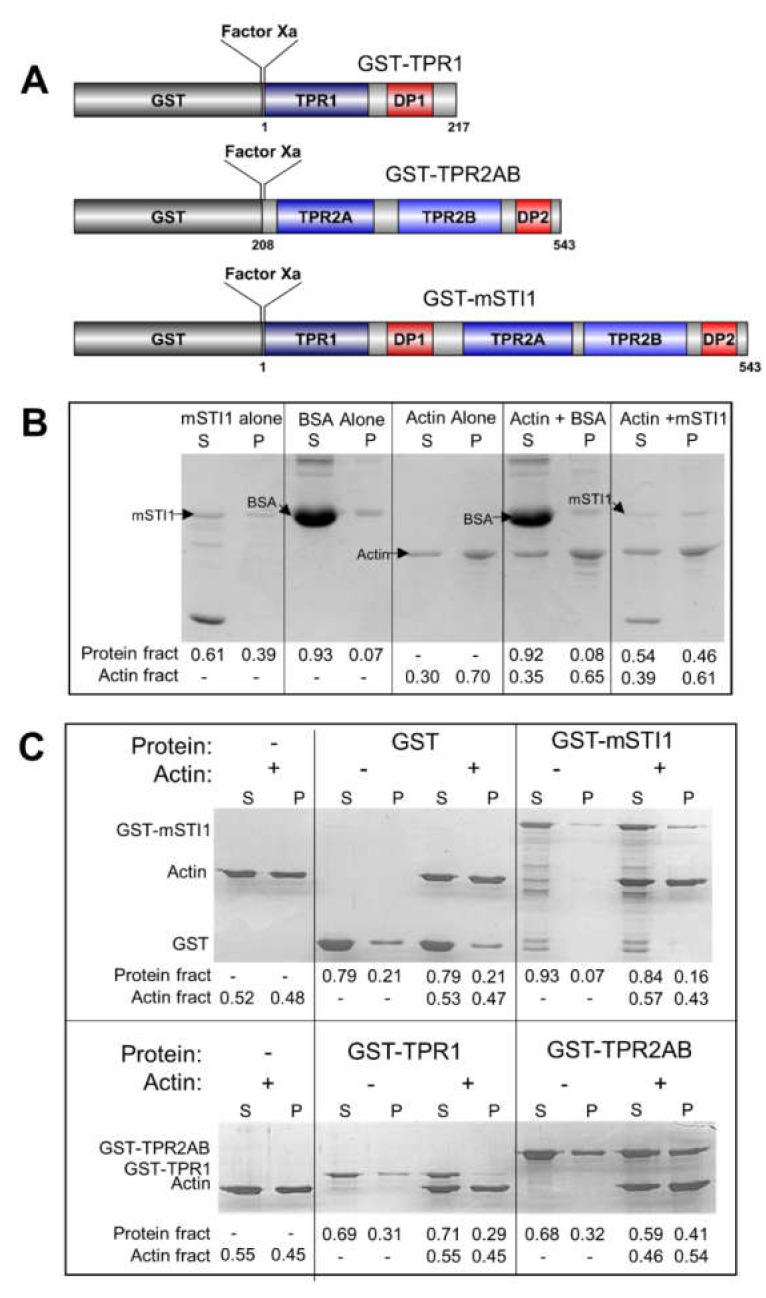
mSTI1 interacts with F-actin. (**A**) Schematic diagram of the recombinant GST-tagged mSTI1 proteins used. Factor Xa represents a cleavage site between the GST-tag and mSTI1. (**B**) Coomassie-stained SDS-PAGE gel of F-actin co-sedimentation assay with mSTI1. (**C**) Coomassie-stained SDS-PAGE gel of co-sedimentation assay to detect F-actin and interaction with full-length mSTI1 or TPR1 and TPR2AB domains. The annotations Actin fract and Protein fract indicate the relative fraction of actin or test protein (GST, BSA, mSTI1 or mSTI1 domains) in the supernatant or pellet, respectively. Data shown are representative of three independent experiments.

There is increasing evidence linking STIP1 and cell migration [56,57,59,60]. STIP1 knockdown resulted in reduced formation of pseudopodia as well as decreased cell migration in a breast cancer cell line, where it also colocalized with actin [59]. Actin is an important cytoskeletal component making up the microfilaments involved in migration. We used a co-sedimentation assay adapted from Srivastava and Barber to evaluate binding of murine STI1 (mSTI1) to filamentous actin (F-actin) [61]. The pairwise amino acid sequence alignment of the mSTI1 and human STIP1 (STIP1) sequences showed a sequence identity and similarity between STIP1 and mSTI1 of 97.4% and 98.9%, respectively (Figure S1). We have determined that mSTI1 can bind Hsp70 and Hsp90 from human cell lysates, suggesting that mSTI1 can participate in similar interactions to STIP1 (unpublished observations). STIP1 contains three tetratricopeptide (TPR) repeat domains (TPR1, TPR2A, and TPR2B) involved in protein–protein interactions, and two aspartate-proline (DP) domains (DP1 and DP2) involved in client activation [62,63,64,65] (Figure 1A). In the assay, F-actin and any associating proteins are distributed to the pellet upon centrifugation, while soluble globular (G)-actin remains in the supernatant. To test whether purified recombinant mSTI1 (Figure S2) bound to F-actin, mSTI1 was combined with G-actin prior to polymerisation. Using densitometry, we quantified the distribution of either actin or the test proteins between the supernatant (S) and pellet (P) fractions relative to the total amount. When actin was incubated alone, the protein was equally distributed between the supernatant and the pellet, indicating the presence of both G-actin (supernatant) and polymerisation of F-actin (pellet). This suggested that the actin used was functional and capable of undergoing polymerisation. The sedimentation profile of actin remained unchanged compared to the profiles when incubated alone, with mSTI1 or with the non-specific protein, BSA, in that G-actin and F-actin pools were identified in the supernatant and pellet fractions, respectively. The proportion of mSTI1 in the pellet was increased when mSTI1 was incubated with actin compared to mSTI1 alone (Figure 1B), suggesting that mSTI1 bound to F-actin. The control protein, BSA, did not change in sedimentation profile when incubated with actin, suggesting that the effect was specific to mSTI1 and not due to the presence of additional protein. To determine which domain of mSTI1 is responsible for binding F-actin, truncated forms of mSTI1 (GST-TPR1 and GST-TPR2AB) and full-length GST-mSTI1 (Figure 1A and Figure S2) were used in the co-sedimentation assay (Figure 1C). Actin, when incubated alone, was found as both G-actin (supernatant) and F-actin (pellet) pools and this distribution was unchanged in the presence of test proteins (Figure 1C). The majority of all GST fusion mSTI1 proteins incubated alone were found in the supernatant with only small quantities found in the pellet, acting as a control to show that these proteins do not sediment alone under the experimental conditions used. In the presence of actin, GST-TPR2AB and full-length GST-mSTI1 showed an increase in the proportion of mSTI1 in the pellet, suggesting an interaction with F-actin (Figure 1C). The control protein GST and GST-TPR1 did not show altered supernatant to pellet distribution when incubated with actin, suggesting that the changes observed with GST-TPR2AB and GST-mSTI1 were specific and not due to additional protein. However, the current analysis is limited by the use of a single concentration of F-actin and future experiments should at least include incubations of a range of concentrations of F-actin alone and with a fixed concentration of mSTI1 in order to assess the interaction in more detail, and to determine whether mSTI1 influences F-actin polymerisation.

**Figure 2:**
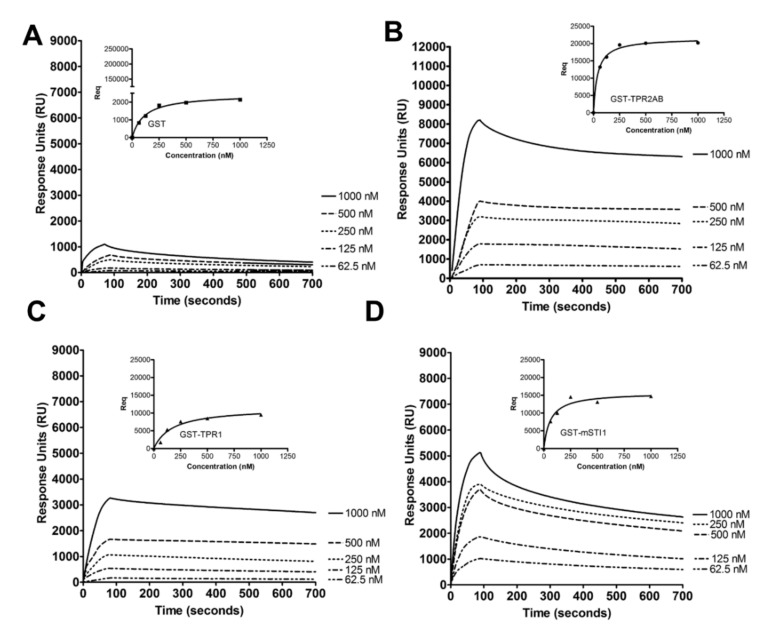
The TPR2AB-DP2 domain of mSTI1 interacts with actin. Surface plasmon resonance (SPR) analysis of binding of (**A**) GST, (**B**) GST-TPR2AB, (**C**) GST-TPR1 and (**D**) GST-mSTI1 to immobilised actin. Data shown are representative of two independent experiments repeated in triplicate. Insets show plots of Req versus mSTI1 concentration.

The interaction between mSTI1 domains and actin was confirmed using surface plasmon resonance (SPR) spectroscopy (Figure 2). Actin was immobilised on the sensor chip surface, while GST, GST-TPR1, GST-TPR2AB, and GST-mSTI1 were flowed over at increasing concentrations. GST-TPR2AB (Figure 2B) showed the greatest binding affinity [K_D_: 33.64 nM] compared to both the full-length GST-mSTI1 (Figure 2D) and GST-TPR1 domain (Figure 2C). The full-length GST-mSTI1 showed a greater binding affinity (K_D_ 55.69 nM) than the GST-TPR1 domain (K_D_ 193.8 nM). While the GST-TPR1 domain bound less strongly than the full-length and GST-TPR2AB proteins, there was noticeable binding of this domain above the GST alone (Figure 2A). The shape of the curves suggested that GST-mSTI1 dissociated from actin more quickly than any of the domains tested (Figure 2D). The co-sedimentation and the SPR analysis indicate that a binding site within the C-terminal TPR2AB-DP2 domains is primarily responsible for the interaction of mSTI1 with actin. The C-terminal regions of STIP1 are common binding sites for a number of interacting proteins, including Hsp90 (which binds primarily to the TPR2A domain) [63,64,66], tubulin (which binds TPR2B) [67], PrPc (which binds between 230 and 245 in TPR2AB domain) [68] and emerin (which binds both TPR2A and B) [69]. Both the truncated versions have been shown to be functional, binding their corresponding murine and human Hsp70 and Hsp90 [70,71]. As such, the inability of TPR1-DP1 to bind F-actin was unlikely a result of misfolded, non-functional proteins. We speculate that the N-terminal portion of mSTI1 has a particular role in actin binding as the TPR2AB-DP2 domains of mSTI1 bound actin strongly and dissociated slowly, whereas inclusion of the TPR1-DP1 domains as part of full-length mSTI1 resulted in faster dissociation. The TPR1-DP1 region alone did not bind actin strongly and thus potentially contributes to faster dissociation of mSTI1 from actin.

### 2.2. Putative Actin-Binding Sites in the STIP1 Sequence

**Figure 3:**
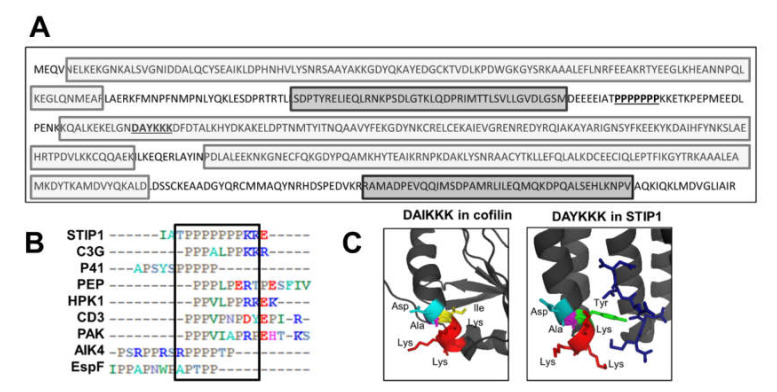
Identification of putative actin-binding sites in STIP1 sequence. (**A**) Amino acid sequence of STIP1. The light and dark shaded boxes show the three TPR domains and the two DP domain sequences, respectively. The underlined residues indicate the polyproline and DAYKKK motif. (**B**) Alignment of the putative SH3-binding motif of STIP1 with other known peptides or proteins that bind SH3 domain, using Clustal Omega software and edited by BioEdit programme. The negatively charged, positively charged and neutral amino acids are shown in red, blue and grey, respectively. The other colours indicate amino acids of similar properties. The conserved polyproline region with basic amino acids is shown with black outline. (**C**) Structure of human cofilin (**left**, PDB:1Q8X) and the TPR2A domain of STIP1 (**right**, PDB:1ELR). Coloured residues represent the canonical and putative actin-binding motifs DAIKKK and DAYKKK, respectively. The residues are indicated as follows: aspartic acid (cyan), lysine (red), alanine (magenta), isoleucine (yellow) and tyrosine (green). Structures were rendered in Pymol.

The human STIP1 (STIP1) amino acid sequence (GenBank AAH02987.1) was analysed for the presence of actin or actin-binding protein-related motifs using Scansite Motif Finder [72] and Motif Scan [73]. Criteria were set to scan for all motifs using a high stringency level and using default parameters, respectively. The two underlined sequences (Figure 3A) show putative actin-binding sites identified using two online motif prediction tools [72,73]. The proline-rich region was identified as a motif that is recognised by Src homology-3 (SH3) domains of actin-binding proteins. SH3 domains are characterised by their protein–protein interactions with proline-rich domains [74]. In some cases, the polyproline region is followed by a region of positively charged, basic amino acids [75], a characteristic present in STIP1 where the polyproline region is followed by two lysine residues (Figure 3B). Clustal Omega software was used to align the putative SH3 polyproline-binding motif of STIP1 with other known peptides or proteins that bind SH3 domains (Figure 3B). In addition to binding to actin-interacting proteins containing SH3 domains, polyproline regions are known to bind actin and play a role in polymerisation [76,77,78,79]. A manual search of the STIP1 sequence identified the residues DAYKKK present in the TPR2A domain that are similar to an actin-binding motif (DAIKKK) of cofilin and the N-terminus of tropomyosin [80]. The cofilin structure (PDB 1Q8X) and STIP1 TPR2A domain structure (PDB 1ELR) were rendered in PyMOL (DeLano Scientific). Figure 3C shows the ribbon structures of human cofilin and the STIP1 TPR2A domain, with coloured residues highlighting the canonical DAIKKK and potential DAYKKK actin-binding motifs, respectively. Both the DAIKKK motif in cofilin and the DAYKKK motif of STIP1 are positioned at the end of an α-helix. Similarity in the orientation of surface-exposed lysine and aspartic acid residues, together with the polar, charged nature of the amino acids, would suggest the residues are available for electrostatic protein–protein interactions and provide support that DAYKKK might represent a putative actin-binding motif. The two putative actin-binding motifs were conserved between the mSTI1 and STIP1 sequences and both were contained within the TPR2AB construct shown to bind actin (Figure 1 and Figure 2). The highlighted proline-rich region in mSTI1 only had one conservative amino acid replacement at the N-terminal residue compared to human STIP1 (Figure S1). Interestingly, polyproline regions have been previously associated with weaker binding than globular domains, as well as enhanced dissociation from the bound proteins [78], consistent with our observation of the enhanced dissociation of STIP1 from actin in the full-length proteins containing the N-terminal region (which includes the polyproline region) compared to TPR2AB alone.

### 2.3. STIP1 Increases Actin ATPase In Vitro but STIP1 Depletion in Cells Reduces F-Actin

**Figure 4:**
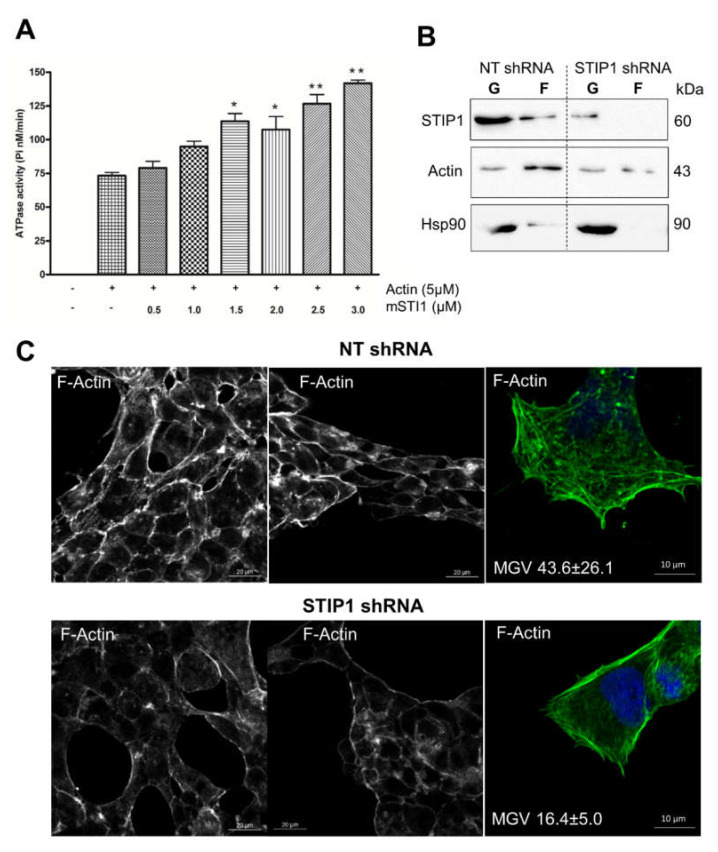
STIP1 regulates actin ATPase and F-actin levels (**A**) ATPase activity using increasing concentrations of mSTI1 with or without actin. Data shown have had the basal ATPase activity of the relevant concentration of mSTI1 alone subtracted. Data are representative of three experiments. Statistical analysis was done using one-way ANOVA and Tukey’s post-test (* *p* < 0.01, ** *p* < 0.001) comparing actin with mSTI1 to actin alone. (**B**) Fractionation of G- and F-actin from HEK293T cells expressing control NT shRNA or shRNA against STIP1. Confocal microscopy in control (NT shRNA) and STIP1-depleted (STIP1 shRNA) HEK293T cells to detect (**C**) F-actin using Actin Green™ 488 Ready Probes (fluorescently conjugated phalloidin). The text in (**C**) indicates the average mean grey value (MGV) ± SD (*n* = 3) of the F-actin signal. Data shown are representative of at least two independent experiments.

ATP hydrolysis by actin is associated with the actin treadmilling process, which in turn controls the length of actin filaments [81]. In particular, ATP hydrolysis is required for the disassembly of actin filaments [9]. To evaluate whether mSTI1 may play a role in actin treadmilling by stimulation of the actin ATPase activity in vitro, increasing concentrations of mSTI1 were incubated with 5 µM actin in a polymerisation buffer containing ATP. The data shown have had the background ATPase activity due to the equivalent concentration of mSTI1 alone subtracted. The actin alone showed ATPase activity above background confirming it was functional (Figure 4A). A dose dependent increase in the rate of ATP hydrolysis by F-actin was observed (Figure 4A). In comparison to F-actin alone, the increase became significant (*p* < 0.01) at mSTI1 concentrations above 1.5 μM. To the best of our knowledge, this is the first report of a protein being able to increase the hydrolysis of ATP by actin. However, the ATPase domains of actin and Hsp70 share structural similarity and STIP1 is known to stimulate the ATPase activity of Hsp70 in yeast [82,83].

**Figure 5:**
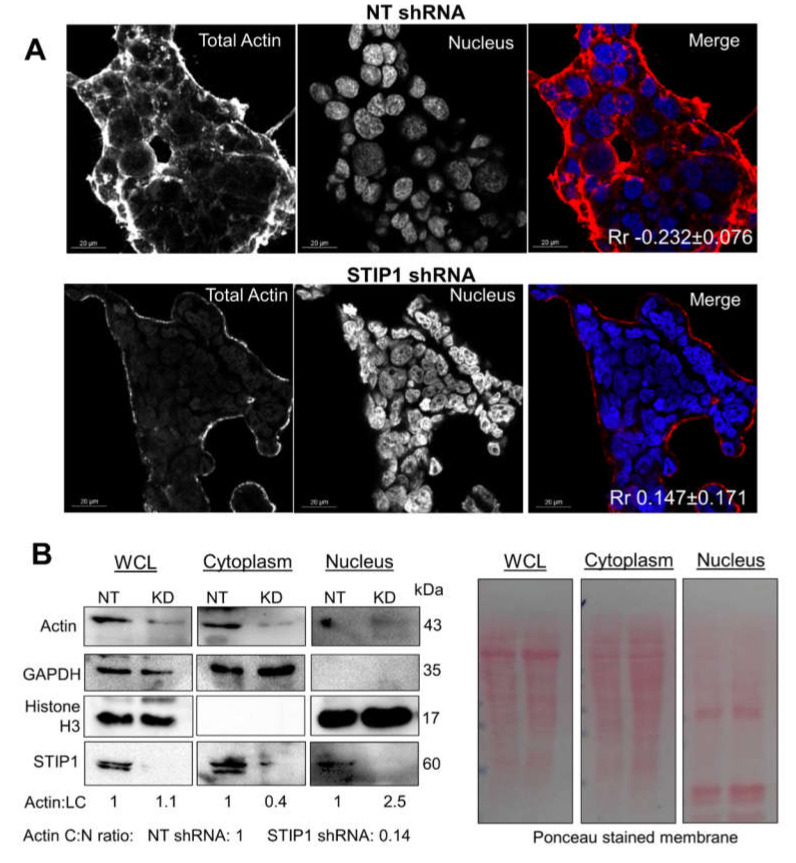
STIP1 regulates actin localization. (**A**) Confocal microscopy in control (NT shRNA) and STIP1-depleted (STIP1 shRNA) HEK293T cells to detect total actin with anti-actin antibody. The text indicates the average Pearson correlation coefficient (±SD, *n* = 3) between the nucleus and actin signals. (**B**) Nuclear-cytoplasmic fractionation and Western blotting of distribution of actin in cells expressing control shRNA or shRNA against STIP1. Actin:LC indicates the densitometry of actin signal to the loading control (GAPDH in WCL and cytoplasm and Histone H3 in nucleus) normalised to the NT shRNA for each fraction. The actin C:N ratio indicates the ratio of the actin:LC signal in the cytoplasm relative to the nucleus normalised to the NT shRNA.

Increasing actin ATPase should result in disassembly of F-actin filaments. We therefore investigated whether STIP1 depletion would affect actin structure and distribution in cells. To validate the in vitro data, we fractionated G- and F-actin from HEK293T cells expressing either control shRNA or shRNA against STIP1 (Figure 4B). G- and F-actin pools were detected in the control HEK293T cell lysates. Hsp90, which is known to bind F-actin in cells, was identified in the F-actin fraction [44]. In the control cells, STIP1 was also identified in the F-actin fraction. Depletion of STIP1 using shRNA led to reduced F-actin and loss of Hsp90 and STIP1 in the F-actin fraction. HEK293T cells expressing non-targeting or STIP1-specific shRNA were subsequently analysed by confocal microscopy for F-actin (Figure 4C). In cells with normal levels of STIP1 (NT shRNA), strong actin staining was detected in the cytoplasm and at cell borders, with low signals in the nucleus (Figure 4C). Consistent with this, cells with depleted levels of STIP1 (STIP1 shRNA) showed little F-actin in comparison to cells with normal levels of STIP1 (NT shRNA) (Figure 4C). The strong F-actin structures around the borders of control HEK293T cells were less predominant and thinner in STIP1-depleted HEK293T cells (Figure 4C). This was particularly seen at the intercellular junctions of cells. To analyse the subcellular localization of actin, we conducted confocal microscopy using an anti-actin antibody and nuclear-cytoplasmic fractionation (Figure 5). Cells with depleted levels of STIP1 (STIP1 shRNA) showed an overall decrease in the actin signal in the cytoplasm, but an increase in the actin signal in the nucleus (Figure 5A), as shown by the Pearson correlation coefficient value (Rr) for the actin and nuclear signals. The Rr represents the correlation of the intensity distribution between two channels, where -1 represents complete negative correlation of the channels and 1 represents perfect correlation [84,85]. However, while there was an increase in the Rr for actin and the nucleus upon STIP1 depletion, the nuclear actin levels were still low and the degree of colocalization between the actin and nuclear signals was only ~15% (Figure 5A). Actin levels have been shown to increase in the nucleus during stress [86], despite the fact that actin lacks a nuclear localization signal (NLS). To confirm the nuclear increase in actin, HEK293T cells expressing or depleted of STIP1 were subject to nuclear-cytoplasmic fractionation (Figure 5B). The controls of histone H3 and GAPDH demonstrated successful nuclear-cytoplasmic fractionation. The total actin levels in the whole cell lysates were not substantially different, but STIP1-depleted cells showed an increase in actin in the nuclear fraction with a concomitant decrease in the cytoplasmic fraction compared to controls which led to a normalised cytoplasmic to nuclear actin ratio of 0.14 compared to 1 for the control cells (Figure 5B). Taken together, the loss of F-actin filaments upon STIP1 depletion was in contradiction to the mSTI1 stimulation of actin ATPase seen in the in vitro analysis. STIP1 is known to stimulate the ATPase activity of Hsp70 in yeast [82], and the ATPase domains of Hsp70 and actin share structural similarity. Therefore, it is possible that while STIP1 may be able to activate the ATPase activity of actin due to structural similarities with the Hsp70 ATPase, this stimulation does not represent the physiological situation in cells. This suggested that the mechanism by which STIP1 regulates F-actin formation in cells may be independent of its ability to stimulate actin’s ATPase activity.

### 2.4. STIP1 Depletion Alters the Ratio of Actin-Binding Proteins Cofilin and Profilin

**Figure 6:**
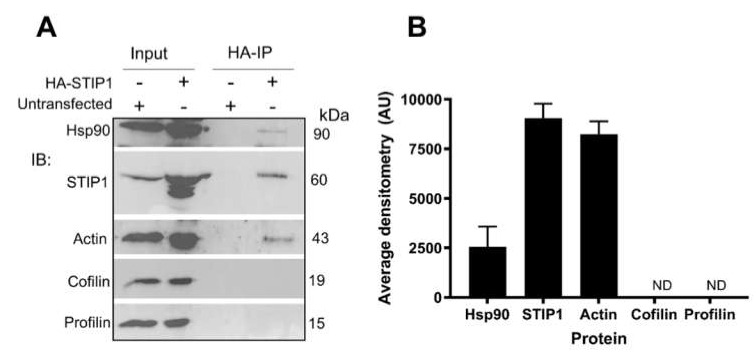
STIP1 and actin, but not cofilin and profilin could be isolated in a common complex. (**A**) Western blotting and (**B**) average densitometry (±SD, *n* = 3) of complexes from HA-immunoprecipitation experiments from untransfected or HA-STIP1-transfected HEK293T cells. ND = not detected.

Actin polymerisation is regulated by numerous protein–protein interactions that promote either filament formation or disassociation. A possible explanation for the apparent contradiction between the in vitro ATPase stimulation data and cell line analysis of F-actin formation is that STIP1 may regulate F-actin formation indirectly through regulation of actin-binding proteins, such as cofilin and profilin. In addition to regulating F-actin formation, these actin-binding proteins regulate actin subcellular localization. Willmer et al. previously showed that STIP1 colocalized with total actin in Hs578T breast cancer cells [59]. Confocal microscopy suggested that STIP1 partially colocalized with actin, cofilin and profilin in Hs578T cells (Figure S3). However, while actin and Hsp90 coimmunoprecipitated with HA-STIP1 from cell lines, neither cofilin nor profilin were detected in these complexes (Figure 6). This suggested that the partial colocalization observed is likely rather due to colocalization of multiple proteins with actin. STIP1 may therefore be indirectly associated with actin-binding proteins through their association with actin. Since STIP1 contains putative actin-binding motifs in its C-terminal region, which also contains the main Hsp90-binding site on STIP1 (TPR2A) [65], it suggests that these complexes are not necessarily mutually exclusive.

**Figure 7:**
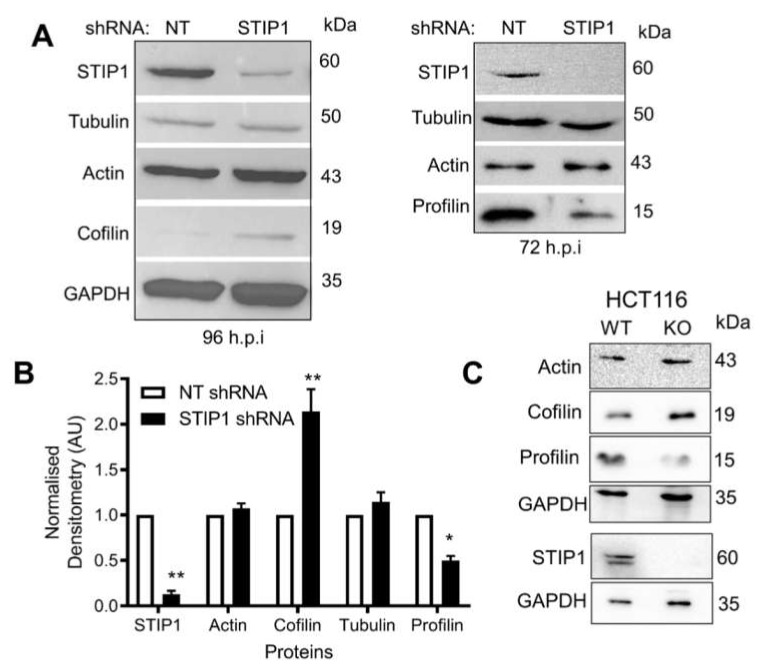
STIP1 depletion upregulated cofilin but downregulated profilin. (**A**) Knockdown of STIP1 in HEK293T cells using STIP1-specific RNA (STIP1 shRNA) and a non-targeting shRNA (NT shRNA) as a control. Lysates were prepared after induction of shRNA by doxycycline for 72 and 96 h followed by Western blotting. (**B**) Densitometric analysis of Western blotting with protein levels normalised to control cell line (NT shRNA). Statistical analysis was done by two-way ANOVA and Bonferroni post-test (* *p* < 0.05, ** *p* < 0.001) comparing shNT versus shSTIP1 lysates. Data are representative of triplicate independent experiments, with graphs showing average densitometry ± SD (*n* = 3). (**C**) Western blotting of protein levels in wild-type and STIP1-knockout HCT116 cells.

Given a possible indirect association between STIP1 and the actin-binding proteins, the levels of cofilin and profilin were analysed by Western blotting using lysates prepared from HEK293T cell lines expressing either non-targeting (NT) or STIP1-specific shRNA (Figure 7A). The densitometry represents average protein levels from replicate analyses of the STIP1-depleted cells normalised to the non-targeting control (Figure 7B). The STIP1 levels were significantly reduced (*p* < 0.01) in the STIP1 shRNA HEK293T cells compared to the non-targeting control. There was a significant reduction in profilin levels (*p* < 0.05) coupled to a significant increase in cofilin levels (*p* < 0.01) in STIP1-depleted cells (Figure 7B). In addition, we analysed the levels of cofilin and profilin in wild-type and STIP1-knockout HCT116 colon carcinoma cell lines. Consistent with the observations in the HEK293T cell lines, the loss of STIP1 resulted in a decrease in profilin levels and an increase in cofilin levels (Figure 7C).

In addition to changes in cofilin and profilin, STIP1 depletion is associated with a loss of RhoC [59], which is specifically involved in regulation of cofilin activity to restrict severing activity at the leading edge or at invadopodia [87]. Therefore, STIP1 depletion would be associated with changes in actin regulating proteins RhoC, cofilin and profilin which should then theoretically culminate in a reduction in actin filaments as observed in our study. In addition, cofilin and profilin regulate the nuclear import and export of actin, respectively [18,39,40]. Enhanced accumulation of nuclear actin in STIP1-depleted cells is therefore consistent with the increase in cofilin levels, combined with reduced profilin levels.

## 3. Materials and Methods

### 3.1. Expression and Purification of GST-Tagged mSTI1 Proteins

GST and GST-tagged mSTI1 fusion proteins containing either full-length protein, or TPR1 and TPR2AB domains were expressed in *Escherichia coli* (*E. coli*) XL1-Blue and purified by GSH-affinity chromatography as previously described [88]. Purification fractions were analysed by SDS-PAGE to confirm successful protein purification (Figure S2). Our purification procedure has previously shown GST-mSTI1 to be over 95% pure by FPLC. The additional bands in the full-length GST-mSTI1 lanes are due to degradation of the full-length protein during SDS-PAGE.

### 3.2. SDS-PAGE and Western Blotting

Samples were analysed by SDS-PAGE according to the standard modifications of the protocol described by Laemmli (1970) [89]. Protein separation using 12% (*v*/*v*) or 14% (*v*/*v*) resolving gels was followed by Coomassie staining or Western blotting according to an adaption of the method of Towbin et al. (1979) [90]. Membranes were blocked using 5% (*w*/*v*) milk powder and incubated overnight at 4 °C with primary antibodies against Hsp90 (1:1000), STIP1 (1:10,000), actin (1:1000), cofilin (1:500), profilin (1:1000), tubulin (1:2500) or GAPDH-HRP (1:5000). The α-STIP1 (Ab126724), α-cofilin (Ab42824), α-tubulin (Ab78078) and α-GAPDH-HRP (Ab185059) antibodies were purchased from Abcam (Cambridge, UK); α-GST (sc-459) and α-Hsp90 antibodies (sc-13119, sc-1055) were purchased from Santa Cruz Biotechnology (Dallas, TX, USA); the α-actin (A2103) and α-profilin (3237S) were obtained from Sigma-Aldrich (St. Louis, MO, USA) and Cell Signalling Technology (Danvers, MA, USA), respectively. Incubation with species-matched HRP-conjugated secondary antibodies was performed for 2 h at room temperature. Densitometry was determined using ImageJ software 1.50i (NIH freeware).

### 3.3. F-Actin Formation, Co-Sedimentation and Bundling Assay

Polymerised F-actin (rabbit muscle, Cat #: P5204 Abnova) was prepared by resuspension (1 mg/mL) in cold Buffer G (5 mM Tris-HCl, pH 8.0, 0.2 mM CaCl_2_), addition of polymerisation buffer (100 mM Tris-HCl, pH 7.5, 500 mM KCl, 20 mM MgCl_2_, 10 mM ATP) and incubation for 1 h at 25 °C. The high-speed co-sedimentation protocol was adapted from that described by Srivastava and Barber [61]. Briefly, 20 µg of GST, GST-TPR1, GST-TPR2AB and GST-mSTI1 or untagged mSTI1 was added to 20 µg of polymerised F-actin or buffer F (90 % *v/v* buffer G, 10 % *v/v* polymerisation buffer) as a control. Following incubation at 25 °C for 30 min, reactions were centrifuged (150,000× *g*) at 25 °C for 1.5 h. For F-actin formation assay, actin (20 µg) resuspended in buffer G was incubated with or without an equal amount of full-length mSTI1 or BSA (20 µg). Addition of polymerisation buffer was followed by incubation at 25 °C for 30 min and high-speed centrifugation (150,000× *g*) at 25 °C for 1.5 h to detect formation of actin filaments. Detection of actin bundling capabilities (low speed co-sedimentation assay) was carried out identically to that described for filament formation except that centrifugation was at 14,000× *g* for 1 h. Supernatant and pellet fractions were analysed by SDS-PAGE with Coomassie staining. The densitometry of protein bands was measured using ImageJ software. The relative fraction of actin or test proteins in the supernatant or pellet fractions was quantified from the densitometry using the equations S/(S + P) or P/(S + P), respectively.

### 3.4. ATPase Assay

Actin (5 µM) was tested for ATPase activity at increasing concentrations of full-length mSTI1 (0.5–3.0 µM) using the EnzChek phosphate assay kit according to the manufacturer’s instructions. Reactions were initiated with MgCl_2_ (2 mM). Addition of CaCl_2_ (50 mM) and ATP (0.5 mM) initiated polymerisation and ATP hydrolysis. Controls included no test protein but included the substrate, ATP. UV absorbance was used to determine the rate of inorganic phosphate (P_i_) production. The linear rates for each of the reactions to the same time point were calculated and compared. Data shown are normalised for background levels of ATPase activity for mSTI1 alone at each concentration.

### 3.5. Surface Plasmon Resonance Spectroscopy

All reactions were carried out using a ProteOn™ XPR36 Protein Interaction Array System (Bio-Rad) (25 °C; Running Buffer: 40 mM HEPES-NaOH, 150 mM KCl, 5 mM MgCl_2_, pH 7.4). The ProteOn™ GLM Sensor chip (Cat #: 176-5012, Bio-Rad, Hercules, CA, USA) was initiated using 50% (*v*/*v*) glycerol and preconditioned using 0.05% (*w*/*v*) SDS and 100 mM HCl at 30 µL/min with successive 60 μL pulses in the horizontal and vertical directions. After which, a pulse each with a volume of 150 μL comprising a 1:1 mixture of EDAC and Sulfo-NHS at 30 μL/min was used to activate the GLM chip surface. Actin (rabbit muscle, Cat #: P5204, Abnova, Heidelberg, Germany) at concentrations of 10 and 100 µg/mL in phosphate buffered saline (PBS) was immobilised on two ligand channels at levels equivalent to ±800 RU and ±8000 RU, respectively, in 10 mM sodium acetate, pH 4.5. Free amines were blocked on a third ligand channel using 1 M ethanolamine and used as an inline reference. GST, GST-TPR1, GST-TPR2AB and GST-mSTI1 sensograms were collected as a 100 μL/min injection for 90 s followed by a 600 s delay where dissociation was monitored. The chip was regenerated by an 18 s pulse injection of 10 mM Tris, pH 8.0, 3 M guanidine-HCl. Duplicate injections were performed for each concentration (5–1000 nM), and blank buffer injections were used as a double reference subtraction. Data analysis was completed using BIA evaluation 4.1.1 (GE Healthcare) and Prism 4 (Graphpad Software).

### 3.6. Maintenance of Cell Lines

The Hs578T breast cancer cell line (HTB-126) was obtained from ATCC maintained in DMEM with 10% (*v*/*v*) FBS, 2 mM l-Glutamine, 100 U/mL PSA and 2 mM insulin at 37 °C, with 9% CO_2_. Non-targeting control (NT shRNA HEK293T) and STIP1 shRNA HEK293T stable cell lines were generated using a doxycycline inducible pTRIPZ plasmid expressing a non-targeting short hairpin RNA (shRNA) or shRNA against STIP1 (STIP1 shRNA). The NT and STIP1 shRNA HEK293T cell lines were maintained in DMEM with 10% (*v*/*v*) FBS, 2 mM l-Glutamine, 0.1 mM NEAA, 1 mM sodium pyruvate, 100 U/mL PSA, 500 µg/mL G418 and 2 µg/mL puromycin at 37 °C, with 9% CO_2_. The shRNA expression was induced with doxycycline (1 µg/mL) every 24 h for 72 or 96 h. HCT116 wild-type and STIP1-knockout cells were a gift from Didier Picard (University of Geneva, Geneva, Switzerland) and were cultured in DMEM with 10% (*v*/*v*) FBS, 2 mM l-Glutamine, 100 U/mL PSA and 1 mM sodium pyruvate at 37 °C, with 9% CO_2_.

### 3.7. Immunofluorescence and Confocal Microscopy

Cells were seeded onto 0.1% (*w*/*v*) gelatin-coated coverslips and shRNA expression induced for 72 h using 1 µg/mL doxycycline. Cells were serum starved in Opti-MEM^®^ Reduced Serum Medium for 30 min and actin polymerisation stimulated by replacement with normal complete medium for 15 min. Cells were fixed in ethanol, permeabilised using 0.1% (*v*/*v*) Triton-X100 in PBS and blocked with 1% (*w*/*v*) BSA/TBS-T (1% *v/v* Tween-20 in TBS) for 45 min. Cells were incubated with primary antibodies (1:100) overnight at 4 °C followed by incubation with fluorescently conjugated species-matched secondary antibodies (1:500) for 1 h at room temperature. Cells requiring F-actin staining were incubated for 30 min with Actin Green™ 488 Ready Probes^®^ Reagent (Cat #: R37110, Life Technologies). Hoechst 33,342 (1 µg/mL) was used to visualise cell nuclei. Data were analysed in Zen software or using ImageJ (NIH).

### 3.8. Transfection and HA-Immunoprecipitation

HEK293T were transfected with the pcDNA-HA-STIP1/HOP plasmid (synthesised by Genscript, USA and which expresses HA-tagged STIP1) using a 1:1 or 1:2 (µg DNA: µL transfection reagent) ratio of XtremegeneHP transfection reagent in 100 μL of serum free Opti-MEM according to manufacturer’s instructions. Untransfected cells were included as a control. Co-immunoprecipitation (IP) of STIP1 associated proteins was achieved using the HA co-IP kits from Sigma-Aldrich according to the manufacturer’s instructions and analysed by SDS-PAGE and Western blotting.

### 3.9. Fractionation of G- and F-Actin from Cell Lysates

Fractionation of G- and F-actin from cell lysates was conducted using the protocol described by Posern and colleagues [91]. HEK293T shNT and shSTIP1 cells were treated with doxycycline for 96 h to induce STIP1 depletion. Equal cell numbers were allowed to adhere overnight, after which they were scraped into buffer (50 mM NaCl, 1 mM EDTA, 0.5% Triton X-100, 20 mM HEPES, pH 7.9) and homogenized using a dounce homogenizer. The homogenate was cleared at 350× *g* for 5 min to remove intact cells, and supernatant subjected to centrifugation at 100,000× *g*, 1 h, 37 °C to separate F-actin (pellet) from G-actin (supernatant). Proteins were detected in the different fractions by Western blotting.

### 3.10. Cytoplasmic and Nuclear Fractionation

HEK293T shNT and shSTIP1 cells were treated with doxycycline for 96 h and the nuclear and cytoplasmic fractions isolated using the Pierce NE-PER kit (Thermo Scientific, Waltham, MA, USA) Aldrich according to the manufacturer’s instructions. Resulting fractions and whole cell lysates were analysed for protein expression by Western blotting.

### 3.11. Statistical Analysis and Reproducibility

All experiments were conducted a minimum of three times unless otherwise indicated. Statistical analysis was completed using GraphPad Prism 4. One-way ANOVAs were employed with Bonferroni or Tukey’s post-tests (as specified in figure legends).

## 4. Conclusions

Taken together, our data suggest that STIP1 plays multiple roles in actin dynamics and cytoskeletal organization. The loss of actin organization in STIP1 knockdown phenotypes suggests a potential role of STIP1 in the organization of F-actin, which might be by direct interaction of STIP1 with actin or via actin-binding proteins known to affect actin dynamics. Indeed, STIP1 depletion modified localization of actin and levels of cofilin and profilin, a phenotype consistent with the observation of an accumulation of actin in the nucleus and a loss of cytosolic F-actin. These data describing changes in the cytoskeleton by STIP1 provide at least one mechanism to explain the reduction in cell migration upon STIP1 depletion identified by other studies. Future studies will determine whether STIP1 is functioning as a co-chaperone for Hsp70/Hsp90 in this regard.

## Supplementary Materials

Supplementary Materials can be found at https://www.mdpi.com/1422-0067/21/9/3152/s1.
