# Supplementary material for: STIP1/HOP Regulates the Actin Cytoskeleton through Interactions with Actin and Changes in Actin-Binding Proteins Cofilin and Profilin
Source: Int J Mol Sci. 2020 Apr 30;21(9):3152. doi: 10.3390/ijms21093152 (PMC7246624; doi:10.3390/ijms21093152)
Supplement: Supplementary file 1 [file ijms-21-03152-s001.pdf]

International Journal of
Molecular Sciences

MDPI

Article

STIP1/HOP regulates the actin cytoskeleton through interactions with actin and changes in actin binding proteins cofilin and profilin

Samantha Joy Beckley[1], Morgan Campbell Hunter[1], Sarah Naulikha Kituyi[1], Ianthe Wingate[1], Abantika Chakraborty[1], Kelly Schwarz[1], Matodzi Portia Makhubu[1], Robert Pierre Rousseau[1], Duncan Kyle Ruck[1], Jo-Anne de la Mare[1,2], Gregory Lloyd Blatch[1,3], Adrienne Lesley Edkins[1,2]*

[1] Biomedical Biotechnology Research Unit, Department of Biochemistry and Microbiology, Rhodes University, Grahamstown

[2] Centre for Chemico- and Biomedicinal Research, Rhodes University, Grahamstown, 6140, South Africa

[3] The Vice Chancellery, The University of Notre Dame Australia, Fremantle, Western Australia, Australia

* Correspondence: a.edkins@ru.ac.za

Supplementary Marterials

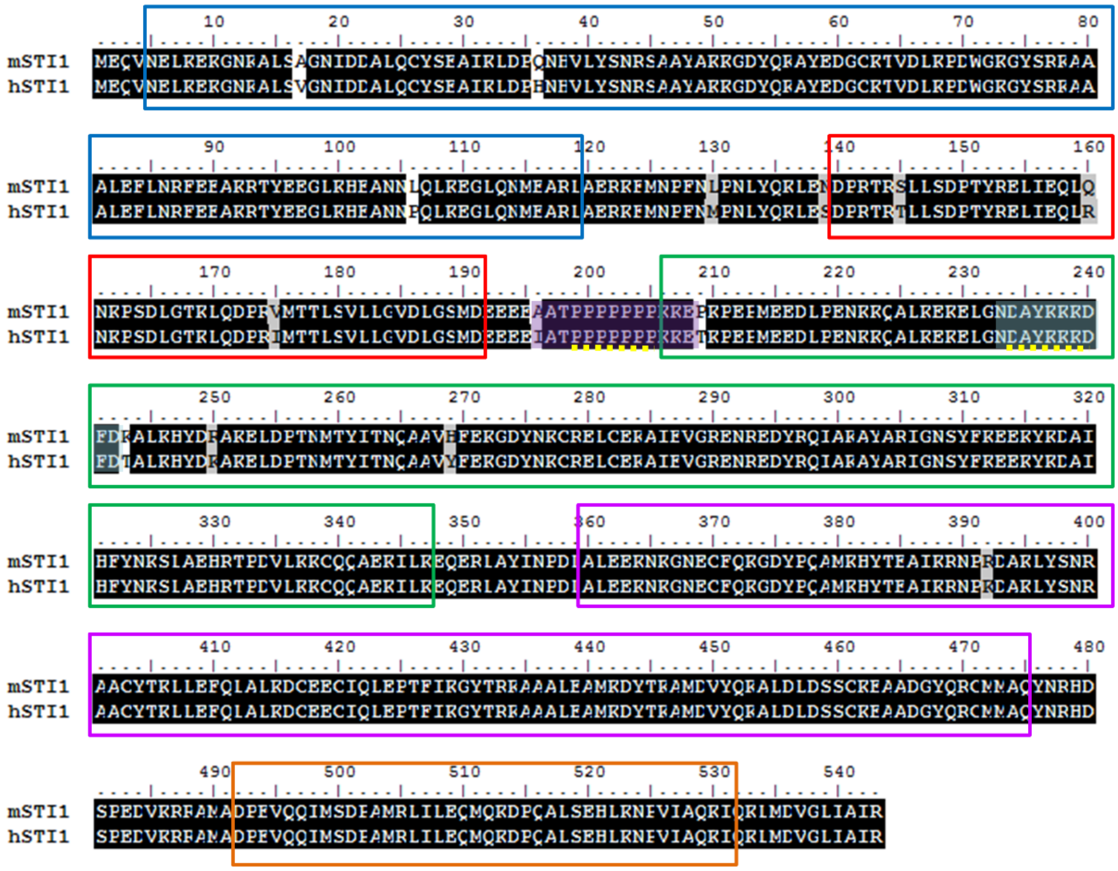

Figure S1. Pairwise sequence alignment of STIP1 protein from *Homo sapiens* and *Mus musculus.* Amino acids with black shading represent identical residues and those with grey shading are considered to be similar. The blue, green and magenta boxes represent the TPR1, TPR2A and TPR2B domains, respectively. The red and orange boxes represent the DP1 and DP2 domains, respectively. The dashed yellow lines and purple and grey shading demonstrate the putative sites for actin binding. hSTI1 (STIP1) amino acid sequences were obtained for *Homo sapiens* (STIP1; Database ID: GenBank

CAG38750.1) and *Mus musculus* (mSTI1; Database ID: GenBank AAH03794.1) and aligned using the alignment tool MAFFT set to default parameters. BioEdit software was used to analyse the similarity and percentage identity.

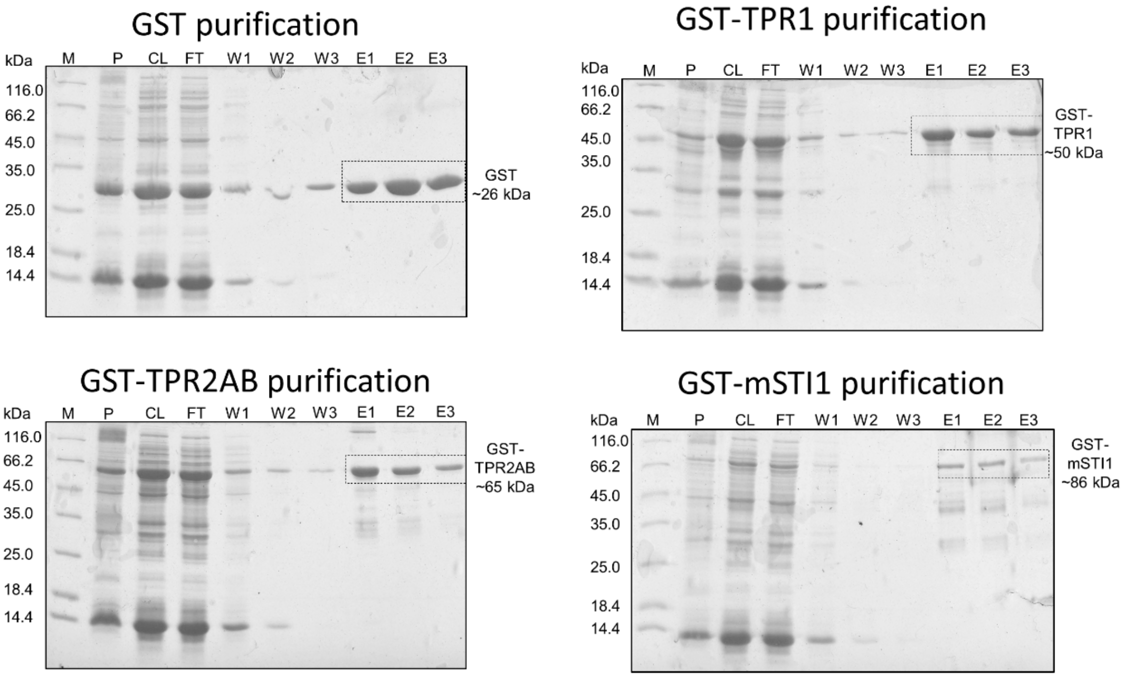

Figure S2. Purification of GST and GST-mSTI1 fusions from bacterial lysates by affinity chromatography. SDS-PAGE analysis of purification of GST, GST-TPR1, GST-TPR2AB and GST-mSTI1 (full length) from BL21 lysates by GSH-affinity chromatography. M: marker; P: pellet (insoluble fraction); CL: cleared lysates (soluble fraction); FT: flow through (unbound to beads); W1-3: washes 1-3; E1-3: elutions 1-3.

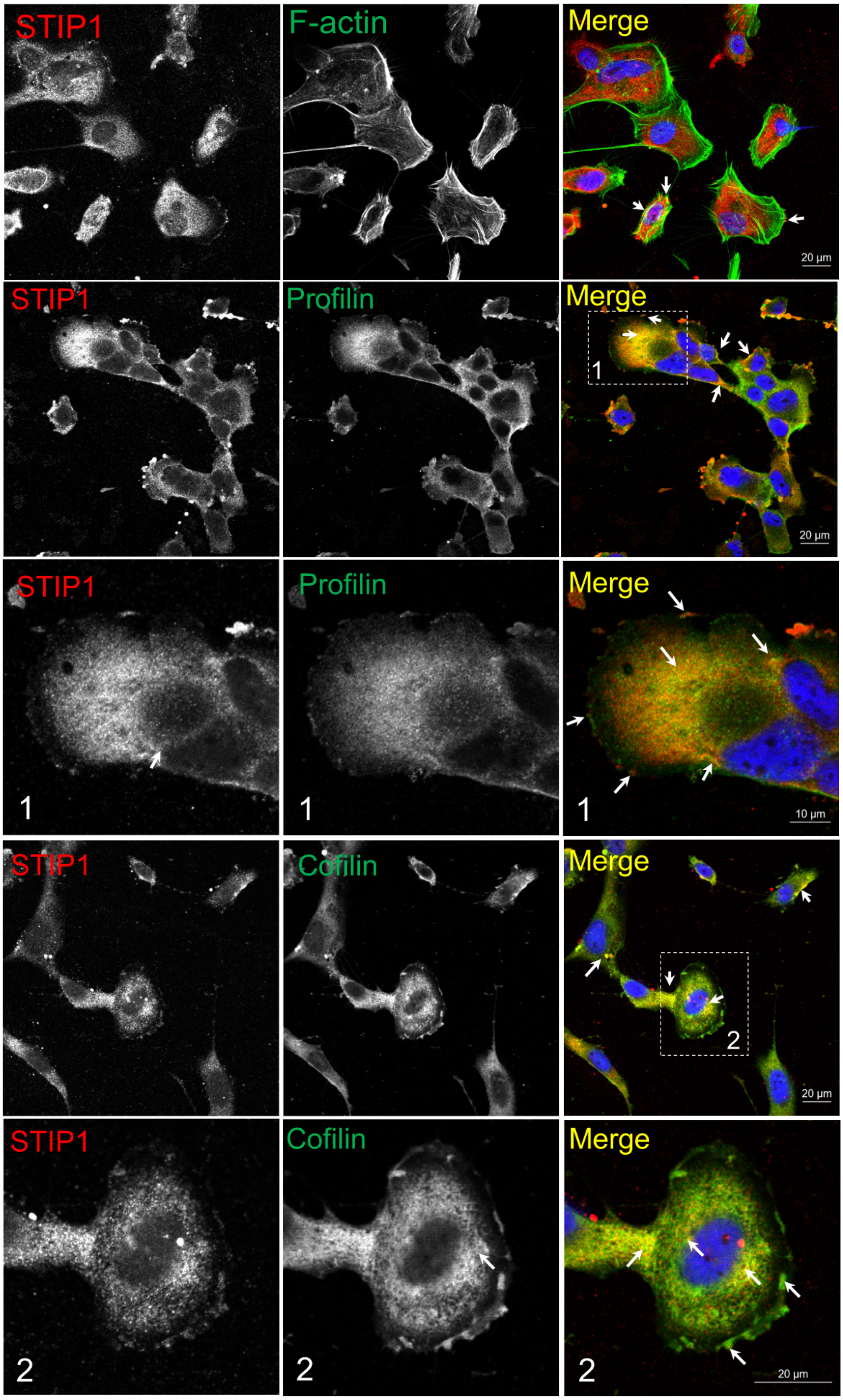

Figure continued over page

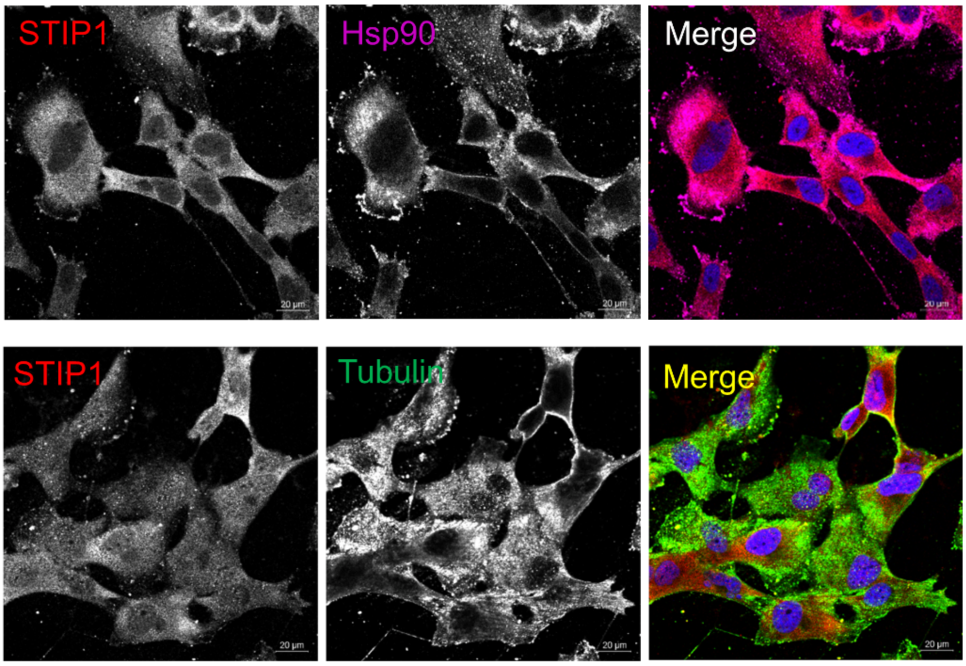

Figure S3. STIP1 colocalises with cytoskeletal proteins. Confocal microscopy of STIP1 (red) and actin (green), profilin (green), cofilin (green), Hsp90 (purple) and tubulin (green) in Hs578T cells. Hoechst 33342 (blue) was used for nuclear staining. White arrows show areas of colocalization. Data are representative of triplicate fields of equivalent cell numbers.
